# A case report of a malignant melanoma in the cardiologic diagnostic workup

**DOI:** 10.1093/ehjcr/ytae312

**Published:** 2024-07-02

**Authors:** Constantin Jahnke, Robert Gramlich, Christian Sander, Stephan Willems, Da-Un Chung

**Affiliations:** Department of Cardiology and Critical Care Medicine, Asklepios Klinik St. Georg, Lohmühlenstraße 5, 20099 Hamburg, Germany; German Centre for Cardiovascular Research DZHK, Partner Site Hamburg, Hamburg, Germany; Department of Cardiology and Critical Care Medicine, Asklepios Klinik St. Georg, Lohmühlenstraße 5, 20099 Hamburg, Germany; Eduard-Arning-Klinik for Dermatology & Allergology, Asklepios Klinik St. Georg, Hamburg, Germany; Department of Cardiology and Critical Care Medicine, Asklepios Klinik St. Georg, Lohmühlenstraße 5, 20099 Hamburg, Germany; German Centre for Cardiovascular Research DZHK, Partner Site Hamburg, Hamburg, Germany; Department of Cardiology and Critical Care Medicine, Asklepios Klinik St. Georg, Lohmühlenstraße 5, 20099 Hamburg, Germany; German Centre for Cardiovascular Research DZHK, Partner Site Hamburg, Hamburg, Germany

**Keywords:** Echocardiography, Cardiac mass, Cardiac tumour, Cardiac malignancy, Malignant melanoma, Case report

## Abstract

**Background:**

Cardiovascular imaging plays an important role in identifying pre-existing cardiac comorbidity prior to the decision on cancer therapy and serves as a reference for detecting changes during treatment and long-term follow-up and also in the further identification of a possible cardiac manifestation of the underlying oncological disease.

**Case summary:**

We report the case of an 81-year-old patient with a malignant melanoma. The patient initially was presented before the start of adjuvant therapy with serine/threonine-protein kinase B-Raf/mitogen-activated extracellular signal-regulated kinase inhibitors. Cardiologic staged diagnostics using transthoracic echocardiography, transoesophageal echocardiography, and cardiovascular magnetic resonance imaging (CMR) revealed with a high probability a cardiac manifestation of the underlying disease. The echocardiographic and CMR results as well as the diagnostic workup are presented.

**Discussion:**

Cardiac masses in general have a variety of differential diagnoses. Cardiac metastases are much more common than primary neoplasms in a ratio of about 10:1. Cardiovascular risk stratification is recommended in all patients with cancer before starting potentially cardiotoxic anticancer therapy. Cardiovascular imaging plays an important role for baseline risk stratification but is also the leading diagnostic tool in the differential diagnosis of cardiac tumours and the planning of a potential therapy.

**Learning points**

- Cardiovascular imaging plays an important role in identifying pre-existing cardiac comorbidity prior to the decision on cancer therapy and serves as a reference for detecting changes during treatment.
- Cardiovascular imaging is the reference method in identification of a possible cardiac manifestation of the underlying oncological disease.
- Malignant melanoma is an aggressive neoplasm that can metastasize to many organs.
- The diagnosis of melanoma with cardiac metastasis before death and without autopsy is rare.
- Secondary neoplasms, such as cardiac metastases, are much more common than primary neoplasms in a ratio of about 10:1.
- Multi-modal approach helps to specify the differential diagnoses and to initiate an aetiology-guided therapy.

## Introduction

Cardiovascular imaging plays an important role in identifying pre-existing cardiac comorbidity prior to the decision on cancer therapy and serves as a reference for detecting changes during treatment and long-term follow-up and also in the further identification of a possible cardiac manifestation of the underlying oncological disease. Malignant melanoma is an aggressive neoplasm that can metastasize to many organs. Data from autopsy study showed cardiac involvement in up to two-thirds of the cases.^1^ The diagnosis of melanoma with cardiac metastasis before death and without autopsy, however, is rare.^2^ Here, we report the case of an 81-year-old patient with a malignant melanoma who was presented for cardiologic diagnostic before the start of adjuvant immunotherapy with serine/threonine-protein kinase B-Raf (BRAF)/mitogen-activated extracellular signal-regulated kinase (MEK) inhibitors.

## Summary figure

Role of multi-modal cardiac assessment of cardiac masses

**Figure:**
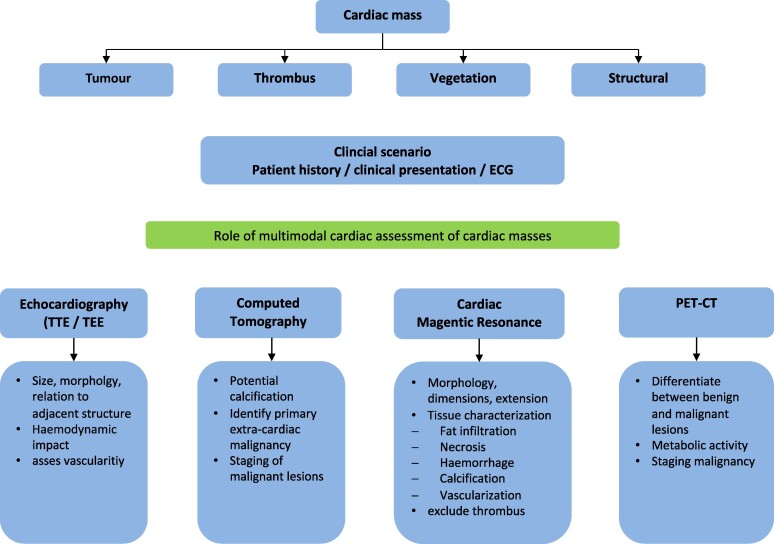


## Case presentation

One year prior to cardiologic presentation, the 81-year-old patient was initially diagnosed with malignant melanoma in the right hip area. Excision was performed with a wide safety margin [tumour stage at initial diagnosis pT4b cNlc MO, clinical stage IV (AJCC 2017) BRAF-positive/NRAS-negative]. In the following year, further suspicious cutaneous findings were excised supraumbilically on the left in the area of the pubic bone and periumbilically on the right, which were revealed to be subcutaneous metastases.

Before the initiation of adjuvant therapy with BRAF/MEK inhibitors, the patient was presented to our outpatient clinic.

On presentation, the patient reported freedom from any symptoms with stable physical capacity. There were no restrictions in everyday life. The patient reported an unremarkable cardiopulmonary history without signs of cardiac decompensation, angina pectoris complaints, or symptomatic cardiac arrhythmia. There was no long-term medication on presentation. The electrocardiogram was inconspicuous, with a normofrequent sinus rhythm without further pathological findings.

A transthoracic echocardiography (TTE) was performed. The results showed a globally normal systolic left ventricular (LV) function with mild LV hypertrophy. Right ventricular function was slightly impaired; the tricuspid annular plane systolic excursion reduced with 14 mm. There was also a minor pericardial effusion without signs of haemodynamic relevance.

**Figure 1:**
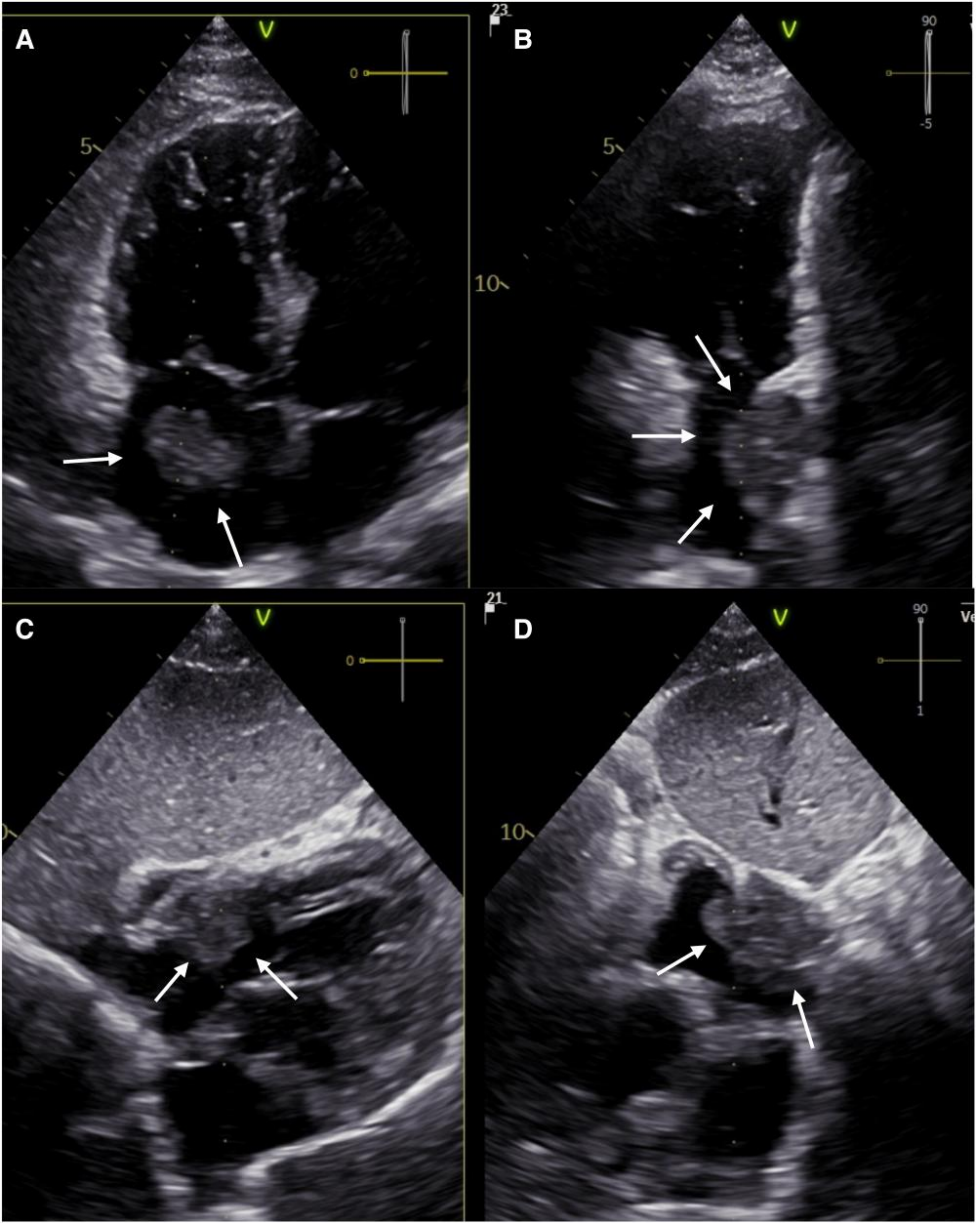
Transthoracic echocardiogram: (*A*) apical four-chamber view demonstrating right atrial mass (arrows), (*B*) corresponding orthogonal plane in the 3D echocardiogram, (*C*) subhypoid plane demonstrating right atrial mass (arrow), and (*D*) corresponding orthogonal plane in the 3D echocardiogram.

A tumour in the area of the right atrium lateral wall (55 × 33 mm) was seen with a suspected borderline expansion (*Figure 1*). Transoesophageal echocardiography (TEE) and cardiac magnetic resonance imaging (MRI) were, therefore, performed for further diagnostics.

**Figure 2:**
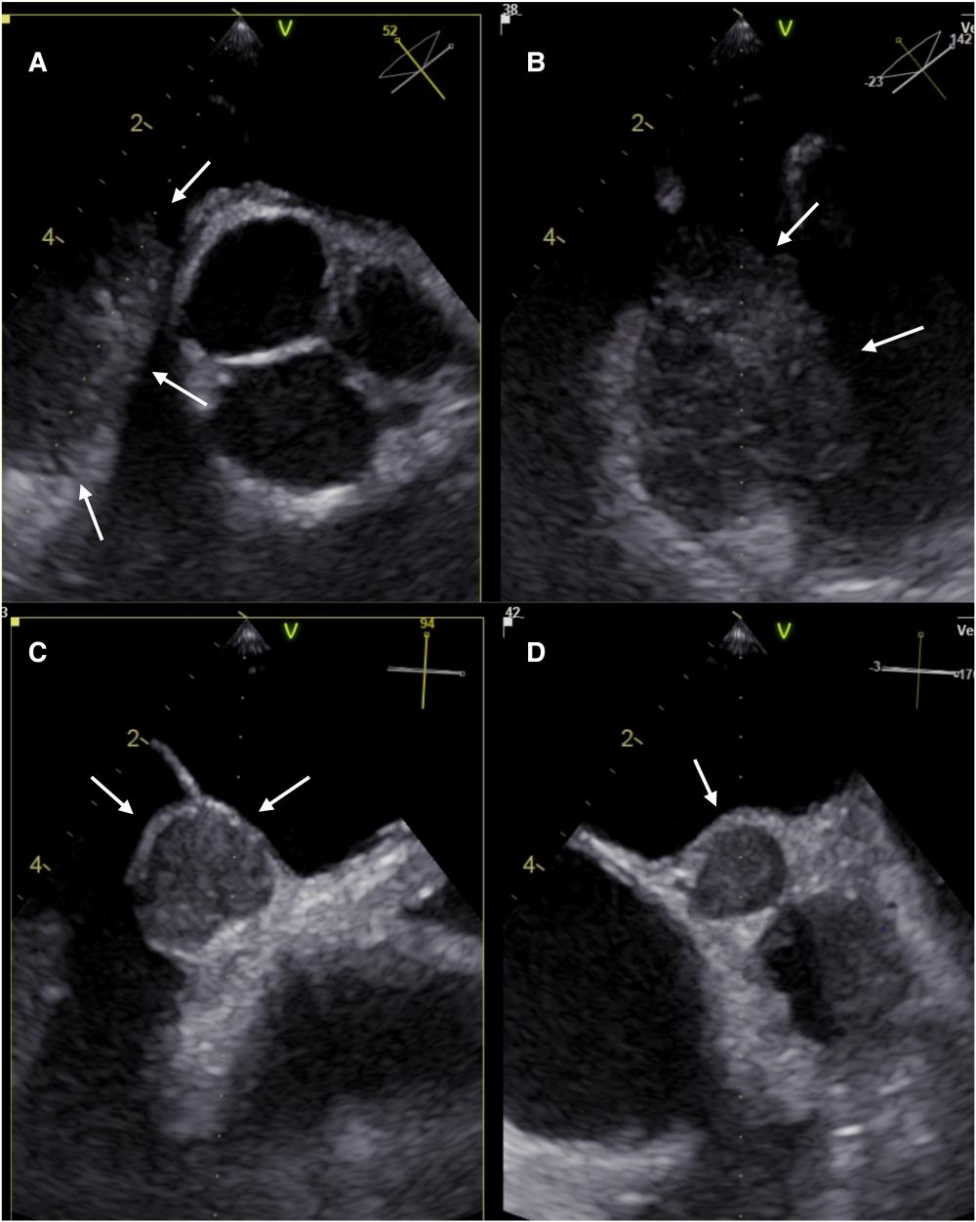
(*A*) Transoesophageal echocardiogram at 52° at the level of the aortic valve demonstrates the cardiac mass in the area of the right atrium (arrows) (*B*) corresponding orthogonal plane in the multi-plane echocardiogram. (*C*) Transoesophageal echocardiogram at 94° demonstrates second encapsulated cardiac mass antero-superior to the atrial septum (*D*) corresponding orthogonal plane in the multi-plane echocardiogram.

Transoesophageal echocardiography revealed a second encapsulated tumorous structure measuring approximately 16 × 16 mm antero-superior to the atrial septum in addition to the original tumour, which was localized in the area of the right atrial appendage (*Figure 2*).

**Figure 3:**
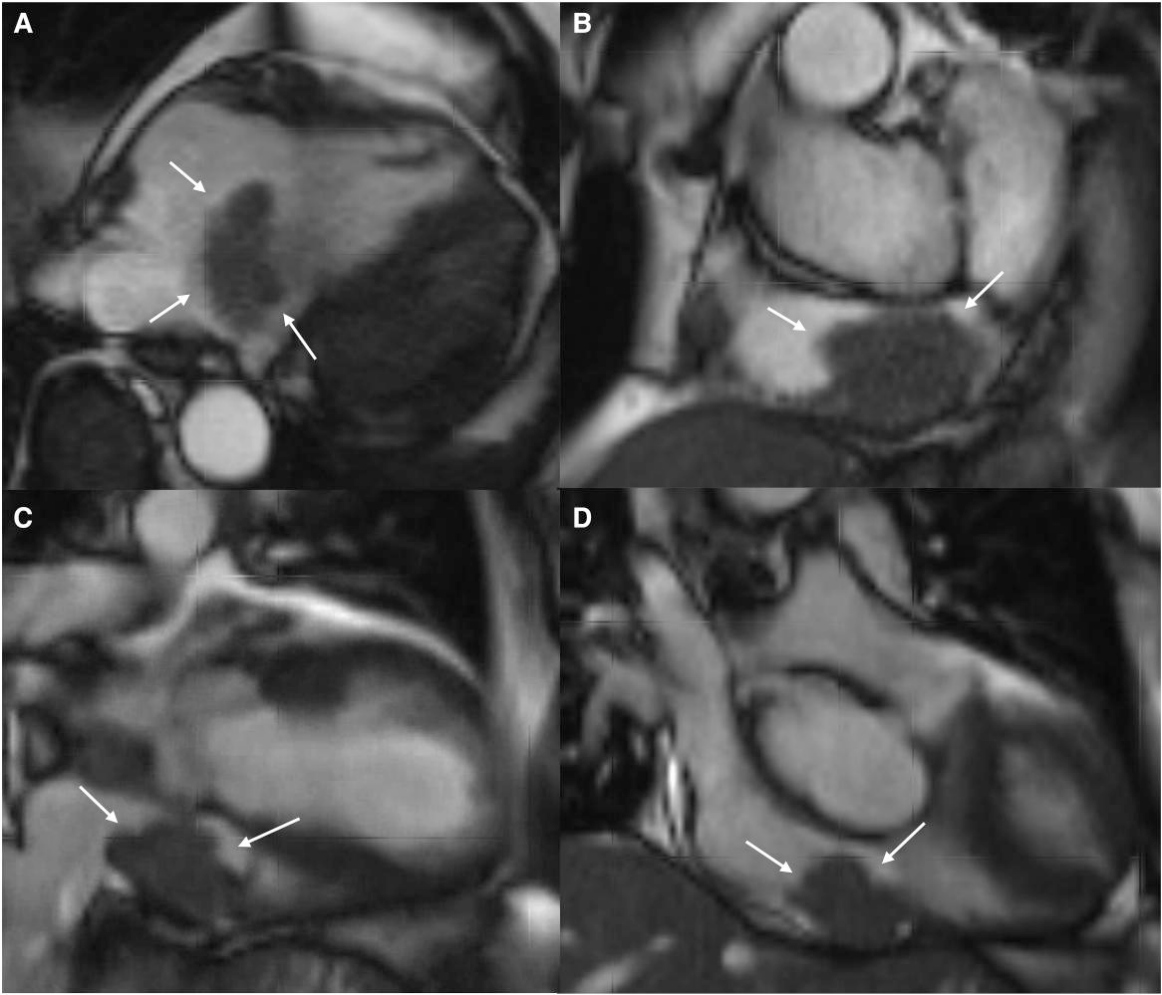
Cardiac magnetic resonance images with a steady-state coherent sequence with balanced gradients demonstrates the cardiac mass in the area of the right atrium (arrows) in (*A*) transversal imaging view, (*B*) short-axis view, (*C*) sagittal view, and (*D*) frontal view.

Cardiac MRI was performed at 1.5 T (Avanto, Siemens Healthineers, Erlangen, Germany). The standard tumour mass protocol includes cine images, T1 and T2 mapping, and late gadolinium enhancement images. As the patient refused to have a contrast agent administered, the images were taken as native images. Cardiac MRI showed an isointense mass in the cine imaging of 48 × 33 × 28 mm in the right atrium with adhesion to the posterior atrial wall and pathologic thickening of the crista terminalis and the atrial septum (*Figure 3*). In the T1- and T2-weighted images, the intra-cardiac findings appeared hyperintense. Unfortunately, mapping values are not available for the scanner that was used. Additionally, multiple nodules of 8, 10, and 13 mm in the anterior pericardial fat tissue were described. Further nodules in the right lung, peritoneum, right hemithorax, and right axilla were seen in the overview and planning images.

The patient underwent a new staging including computed tomography (CT) of the thorax and abdomen and an MRI of the cranium, which showed extensive progression of the underlying disease. A subsequent tumour board conference recommended the continuation of treatment with the BRAF/MEK inhibitor and early follow-up care in accordance with the guidelines, taking into account the patient's age and a Grade 2 on the Eastern Cooperative Oncology Group Performance Status Scale. In consideration of the results with multiple metastases and the lack of signs of haemodynamic compromise, an endocardial biopsy was not performed and possible cardiac surgery was considered non-beneficial.

After approximately 3 months of therapy with BRAF/MEK inhibitors, a re-staging including CT of the thorax and abdomen as well as a brain MRI was performed. The findings showed an overall partial remission with completely regressed pulmonary metastases, size-regressed thoracic lymph node metastases, and a reduced number of size-regressed intra-abdominal metastases and size-regressed cutaneous metastases. However, there are also isolated cases of intra-abdominal metastases that are constant in size or progressive in size.

The brain MRI showed no clear evidence of new brain metastases. Previously described lesions were regressive in size compared with the initial image.

A follow-up TTE showed a normal systolic LV function and as far as a transthoracic assessment allowed a size regression of the intra-cardiac findings. On the basis of the findings, treatment with BRAF/MEK inhibitors was continued with further follow-up appointments.

## Discussion

According to the current 2022 European Society of Cardiology (ESC) guidelines on cardio-oncology, cardiovascular risk stratification is recommended in all patients with cancer before starting potentially cardiotoxic anticancer therapy.^3^ Cardiology referral is recommended in high-risk and very high-risk patients before anticancer therapy.^3^

In the context of BRAF/MEK inhibitors, the main cardiovascular side effects to be aware of are hypertension, pulmonary embolism, QTc prolongation, and cancer therapy–related cardiac dysfunction.^4^

Cardiovascular imaging plays an important role in identifying patients with not yet clinically manifest cardiovascular disease and in determining the degree of pre-existing cardiac comorbidity prior to the decision on cancer therapy. Furthermore, it serves as a reference for detecting changes during treatment and long-term follow-up. Transthoracic echocardiography is the preferred imaging modality for baseline risk stratification as it allows quantitative assessment of LV and right ventricular function, single cavity dimensions, assessment of possible hypertrophy, regional wall motion abnormalities, diastolic function, evaluation of valvular and pericardial disease, and the determination of pulmonary arterial pressure.^3,5^

Cardiovascular imaging also serves as a reference for detecting a possible cardiac manifestation of the underlying oncological disease.

Malignant melanoma is an aggressive neoplasm that can metastasize to many organs. Data from an older autopsy study of 70 malignant melanoma patients showed cardiac involvement in up to 64% of the cases.^1^ The diagnosis of melanoma with cardiac metastasis before death and without autopsy is rare, which may be due to the fact that only about 16% of patients have cardiovascular symptoms and cardiac metastases are difficult to detect on conventional staging images.^2^

In general, cardiac masses have a variety of differential diagnoses including primary and secondary neoplasms and non-neoplastic masses such as thrombus, vegetations, calcified lesions, and other rarer diseases. Secondary neoplasms, such as cardiac metastases, are much more common than primary neoplasms in a ratio of about 10:1.^6^

The diagnostic pathway should be based on the epidemiology of the tumour type, for which imaging procedures and, if possible, histopathological diagnosis are necessary. Imaging must support the differential diagnosis of cardiac tumours as well as the evaluation of therapy including cardiac surgery.^6^

The standard examination is TTE, which may be followed by TEE.^7^ Cardiac MRI allows further characterization of the cardiac tumour tissue and further specification of the differential diagnoses.^6^

CT and positron emission tomography (PET) can be helpful in differentiating between malignant and benign lesions and the detection of non-cardiac metastases or primary carcinomas.^8,9^ The multi-modal approach helps to specify the differential diagnoses and to initiate an aetiology-guided therapy.

Investigators should, therefore, take the necessary care to recognize a possible cardiac manifestation of the underlying oncological disease even in the context of a routine examination. This will hopefully increase the rate of detected cardiac metastases of malignant melanoma in the future and might help to improve the outcome.

## Data Availability

All data underlying this article are available as part of the article. No additional source data are required.

## Lead author biography

Constantin Jahnke graduated in medicine at the University of Hamburg with stages in Innsbruck/Austria and Durban/South Africa. From April 2019 to December 2022, his residency was at the Department of Internal Medicine - Cardiology at the German Heart Center Berlin and, since January 2023, at the Asklepios Klinik St Georg in the Department of Cardiology and Intensive Care Medicine. His particular interest is in cardiac imaging.

**Content:** Patient consent for publication of the case was obtained as per COPE guidelines.

## Notes

**Funding:** None declared.
